# Socioeconomic status, urbanicity and risk behaviors in Mexican youth: an analysis of three cross-sectional surveys

**DOI:** 10.1186/1471-2458-11-900

**Published:** 2011-11-30

**Authors:** Juan Pablo Gutiérrez, Erika E Atienzo

**Affiliations:** 1 Division of Surveys, Centre for Evaluation Research and Surveys, National Institute of Public Health, Cuernavaca, Mexico; 2 Health Policy Unit, London School of Hygiene & Tropical Medicine, University of London, London, UK; 3 Division of Reproductive Health, Centre for Research on Population Health, National Institute of Public Health, Cuernavaca, México

## Abstract

**Background:**

The relationship between urbanicity and adolescent health is a critical issue for which little empirical evidence has been reported. Although an association has been suggested, a dichotomous rural versus urban comparison may not succeed in identifying differences between adolescent contexts. This study aims to assess the influence of locality size on risk behaviors in a national sample of young Mexicans living in low-income households, while considering the moderating effect of socioeconomic status (SES).

**Methods:**

This is a secondary analysis of three national surveys of low-income households in Mexico in different settings: rural, semi-urban and urban areas. We analyzed risk behaviors in 15-21-year-olds and their potential relation to urbanicity. The risk behaviors explored were: tobacco and alcohol consumption, sexual initiation and condom use. The adolescents' localities of residence were classified according to the number of inhabitants in each locality. We used a logistical model to identify an association between locality size and risk behaviors, including an interaction term with SES.

**Results:**

The final sample included 17,974 adolescents from 704 localities in Mexico. Locality size was associated with tobacco and alcohol consumption, showing a similar effect throughout all SES levels: the larger the size of the locality, the lower the risk of consuming tobacco or alcohol compared with rural settings. The effect of locality size on sexual behavior was more complex. The odds of adolescent condom use were higher in larger localities only among adolescents in the lowest SES levels. We found no statically significant association between locality size and sexual initiation.

**Conclusions:**

The results suggest that in this sample of adolescents from low-income areas in Mexico, risk behaviors are related to locality size (number of inhabitants). Furthermore, for condom use, this relation is moderated by SES. Such heterogeneity suggests the need for more detailed analyses of both the effects of urbanicity on behavior, and the responses--which are also heterogeneous--required to address this situation.

## Background

The relationship between health risk behaviors and living conditions is a critical issue on which there is little research. An understanding of how risk behaviors are affected by one's social and physical environment could be a key element in the development of effective youth health policies.

The current youth population (15-24 years old) worldwide comprises the largest generation in history [1]. By 2050, Latin American youths will number approximately 83 million [2]. This population growth goes hand-in-hand with the trend of increasing urban populations. In recent decades, there has been a rapid increase in the proportion of people living in urban areas around the world. Among the developing regions, Latin America stands out for its large urban population, accounting for 77% of its total inhabitants [3]. Mexico, which is among the four largest countries in the region, is also at an advanced stage in urbanization transition, with an urban population of 70% [4].

Official statistics indicate that about 46% of Mexicans live in poverty, and 10% in extreme poverty [5]. Furthermore, a recent concern in the country relates to the youth population. Different surveys have shown that about 22% of individuals between 12 and 29 years old are neither in school nor working [6].

Young people constitute a critical group in social transformation, so their welfare has an impact on development. Although they tend to be conceived of as healthy, the consequences of their behaviors are known to surface in the long run [1]. According to the World Health Organization, 70% of premature adult deaths are linked to behaviors established during adolescence, among which are the consumption of addictive substances [7] and sexual risk behaviors.

In Mexico, alcohol and tobacco consumption has risen among youth. According to the most recent National Addictions Survey (ENA 2008), 15% of 12-17 years old reported having smoked, and 10% reported being active smokers. Approximately 11% of males and 7% of females reported being heavy drinkers (5 or more and 4 or more drinks per occasion, respectively), and the cumulative incidence of illicit drug use was 3.7% in males and 2.1% in females [8].

Some consequences of risk behaviors appear before adulthood. For example, a lack of or inconsistent condom use translates into early pregnancies and sexually transmitted infections [9]. While 30% of Mexicans 16-19 years old have initiated sexual activity, only 63% of males and 38% of females use condoms during their first sexual encounter [10].

Abundant literature identifying individual factors underlying risk behaviors has been published, and the findings suggest that the issue should be investigated at more than just the individual level [11,12]. Thus, attention has recently been drawn to contextual variables [13,14].

Urbanization--an increase in the urban share of the total population--and its relation to health have been discussed in the literature, highlighting both the positive effects of having more access to services and regular employment and the negative effect of urbanicity [15]. Still, little is known about the relation between urbanicity and adolescent health risks. On one hand, urbanicity can facilitate access to goods associated with risk behaviors such as tobacco and alcohol. On the other hand, the effects of preventive campaigns are likely to arise more rapidly in urban areas, attenuating participation in risk behaviors.

Understanding the role of urbanicity in health requires exploration of the different dimensions of urban context and social determinants that can both harm and promote behaviors affecting wellbeing [16]. For instance, urbanicity has been linked to social and cultural aspects of the onset of adult life [17]. Traditionally, individuals come of age earlier in rural environments and participate in practices acceptable among adults, such as early marriage [18]. Conversely, urban localities offer youths diverse lifestyle models that may be absent in rural localities, while rural youth may benefit from closer relations that are often rare in urban localities [19].

Likewise, economic factors are intimately linked to population size. For instance, it is well known that rural communities are still beset not only by greater physical and social isolation [20] but also by severe poverty compared with urban communities [19,21]. However, even when poor youth in urban zones surround themselves with services that are unavailable in rural localities, their access to these resources is socially determined [16]. Poor urban youth lack capital to pay for goods such as education, health services and transportation. In addition, they deal with a challenging environment because urban poverty is not only associated with distance from infrastructure or services, but also with social exclusion [22].

While the effects of socioeconomic status (SES) on risk behaviors have been widely documented, few studies have analyzed their interaction with urbanicity, although such a relationship would be expected [23-25].

No universal definition exists in terms of what an urban area is, and definitions vary among countries and over time [25]. In Mexico, the National Institute of Statistics and Geography (INEGI) classifies localities with less than 2,500 inhabitants as rural; the rest are classified as urban [26]. Among urban localities, there is a further categorization, with small urban localities defined as up to 50,000 and metropolitan areas as having more than one million inhabitants [27].

However, a dichotomy of rural versus urban seems limited when identifying features of the environment that may be related to adolescent risk behaviors because individuals from small towns and large cities would be placed in the same category, although behavioral differences related to the setting would be expected to exist [28]. Therefore, a more accurate indicator showing the gradations of urbanicity may be of value [25].

According to previous studies in Mexico, urban residency is linked to regular and daily tobacco consumption among youth [29] and higher odds of drug and alcohol consumption [30], condom or contraceptive use [31,32] and sexual initiation [32]. However, as there is also evidence linking these behaviors to the socioeconomic status of individuals and/or their households, it seems relevant to not only measure the relation between risk behaviors and urbanicity but also to disentangle how SES affects this relation.

To delineate the role of urbanicity in risk behaviors among Mexican youths, this study analyses the relation between locality size, and tobacco and alcohol consumption as well as sexual initiation and condom use, incorporating an SES indicator as a moderating factor. For this we use a large sample of adolescents from low-income households in Mexico. We hypothesize that risk behaviors are associated with locality size and that this association is moderated by socioeconomic status.

## Methods

A cross-sectional study was conducted with information from three different household surveys from rural, small urban and large urban localities (localities are the geographical areas defined as the lowest administrative division in Mexico) in various states of Mexico in 2001 and 2003.

The surveys were part of the regular evaluation of a national social development program called *Oportunidades *(formerly *Progresa*), a conditional cash transfer program in Mexico that seeks to strengthen the capacity of families living in extreme poverty. Program eligibility is defined based on a socioeconomic score that is estimated using socioeconomic and demographic characteristics such as housing materials, assets and demographic structure. The program determines a cut-off point for this score. Thus, households with a score above the cut-off point are considered eligible for the program, and others are considered non-eligible.

*Oportunidades *began in 1997 in rural areas of Mexico and was extended to small urban areas in 2001 and to large urban areas in 2002. Several rounds of surveys have been implemented to gather data regarding health, education and living conditions with the aim of evaluating the program. Surveys include both eligible and non-eligible households from the same areas. Because household eligibility for the program is defined by socioeconomic status, marginalized areas in Mexico are overrepresented in these surveys.

### Sampling procedures and data collection

In rural areas, localities were sampled at two times. First, in 1997 a set of 506 localities from seven states were probabilistically selected for the evaluation, using census information at the local level to identify the most marginalized areas in terms of a high concentration of low-income households. Second, in 2003 a new sample of 151 poor localities was selected from a list of 14,000 rural localities not yet incorporated into the program. A matching procedure was used that allowed for the identification of a group of localities with characteristics similar to those of the beneficiary localities selected in 1997 (506). All households from these 657 localities (506 + 151) were surveyed in 2003. All households from the localities added in 2003 (n = 151) and some households from the other 506 were not in the program when surveyed (mostly new households). To avoid contamination from program effects, the analysis described here includes only those households non-incorporated into the program (i.e. households that were non-beneficiaries of the program at the time of the survey in 2003, regardless of their eligibility).

In 2001, as the program was extended to small urban areas, a survey targeting poor localities between 2,500 and 75,000 inhabitants was implemented. The survey sampled areas with a high concentration of poor households according to information from the 2000 Mexican Census. A probabilistic and stratified sample was used, with stratums based on geographical region, marginality (as the prevalence of poverty in these localities) and population size. A total of 23,000 households in 203 localities were effectively surveyed in the autumn of 2001. Because all of these households were surveyed (regardless of their eligibility for the program) before the program was implemented in their localities, all of them were used for the analysis described herein.

Finally, in 2002, the program applied a reduced form of its targeting algorithm to all urban households in areas of 50,000-1,000,000 inhabitants in Mexico. Households were classified as eligible or non-eligible using information from the census. Clusters of 500 or more eligible households were identified and classified as intervention zones, with the rest classified as non-intervention zones. The program defined which zones should receive the intervention immediately and which ones should receive it later according to their priority and needs. A probabilistic, stratified and clustered sample was selected for evaluation purposes, resulting in 149 blocks of households from the intervention zones. Another sample of 387 blocks from non-intervention areas was selected using matching techniques (to identify blocks with characteristics similar to those of the intervention areas). In all selected blocks, a sample of households was selected using systematic sampling. These households comprised the sample for the 2003 survey targeting urban areas. No households from the non-intervention areas were in the program when surveyed, although some households from the intervention areas were. As in the survey targeting rural areas, only those households not incorporated in the program were included in this analysis (i.e. households that were nonbeneficiaries of the program at the time of the survey regardless of their eligibility).

The details of these surveys are available elsewhere [33-38]. For all three surveys, data were collected at the household level using comparable questionnaires. In addition to the questionnaire for household informants, young household members were interviewed in relation to their education, labor characteristics and health-related behaviors. For this analysis, we only used variables measured in the same way for the three surveys. The following variables were considered for each household: poverty score, state, municipality and locality. Information on the variables of interest was acquired through the youth questionnaires. Adolescents were asked to sign an informed consent declaration. For adolescents under the age of 16, parents were asked to provide consent. The protocols of the three surveys were approved by the Mexican National Institute of Public Health ethics and research committees.

### Data management

We first homogenized the three databases in terms of study variables. A single database was then created with information from all adolescents 15-21 years old from each survey: 7,693 from the rural, 9,199 from the semi-urban and 8,588 from the urban. To exclude possible effects of the program as behavior modifiers, the analysis comprised only information from those households that had not been incorporated in the *Oportunidades *program at the time of the surveys. Thus, after excluding participants who had participated in the program (51% from rural and 29.4% from urban localities), the sample conformed to 3,726 adolescents from rural, 9,199 from semi-urban (no adolescents were excluded from this sample) and 6,064 from urban areas.

The dependent variables were risk behaviors. Adolescents were asked if they had ever smoked, if they currently smoked and if they currently drank alcohol even on an occasional basis. Regarding sexual behavior, they were asked for their age at first intercourse, which was then used to classify adolescents who had had sexual relations and those who had not. Among sexually active adolescents, condom use was assessed in relation to their last sexual encounter.

All the dependent variables were dichotomous with 0 = No and 1 = Yes values. The main exposure variables were locality size and SES. Independent variables included age (*continuous variable*), gender (*0 = female *and *1 = male*), marital status (*0 = single *and *1 = married/in a consensual union*) and educational level (*1 = None; 2 = Elementary school; 3 = Secondary school; 4 = High school; 5 = Technical or professional studies or College*). The reference categories were those with a value of "0" for the dichotomous variables and "No education" for educational level. For SES, we used the household poverty score that determines program eligibility. This score consists of a continuous variable for which higher positive scores denote lower SES (or severe poverty), with 0.69 being the threshold classifying a household as poor. *Oportunidades *uses discriminant analysis within a variety of observed characteristics that are proxies for poverty, such as housing materials, the possession of goods, water and sanitation facilities, education and family structure. This allows one to identify variables that discriminate between poor and non-poor households and compute an index that functions as a rule for classifying households as poor, almost poor (closer to the poverty line) and non-poor. For this document, the poverty index or household SES was classified into quartiles according to the distribution of the scores in the final sample, where the 4th quartile represents the 25% of adolescents living in the poorest households and the 1st quartile the 25% living in the least poor households (reference category). More information is available in the *Oportunidades *methodological documents [37,38].

To classify adolescents according to the size of the localities in which they lived, census data from INEGI were consulted regarding the number of inhabitants per locality. A categorical variable was then created: (1) rural (*≤ 2,500 inhabitants*), (2) small semi-urban (*> 2,500 and ≤ 15,000 inhabitants*), (3) large semi-urban (*> 15,000 and ≤ 100,000 inhabitants*), and (4) urban (*> 100,000 inhabitants*), with rural localities as the reference category. We used this classification because it was the official domain for classifying locality size used in the last census [39].

### Statistical analysis

Participants with missing data were removed from the database (n = 1,015). The final sample consisted of 17,974 adolescents residing in 704 localities. Statistical analyses were conducted using the SVY commands in Stata 10.0 (Stata Corp, College Station, TX), which are complex survey commands that allow for adjustment of standard errors in all clusters of individuals. In the surveys described herein, the clusters were the primary sampling units (localities in the semi-urban and rural zones, and blocks in the urban zones).

The data were first examined to identify differences due to locality of residence. Subsequently, a multivariate logistic regression model that accounted for clustering was adjusted for each dependent variable with age, educational level and an interaction between marital status and gender (as the potential of being married is expected to be different for males and females) as independent variables. Furthermore, in anticipation of any biases resulting from the inclusion of measurements obtained over two different years, a variable was incorporated to identify the year in which the data were collected (0 = 2001 and 1 = 2003).

The main exposure variables were evaluated by introducing an interaction between SES and locality size. Locality size and SES categories, and the interaction between each, were modeled as indicator variables, excluding the reference category. We adopted Ai and Norton's approach for the interpretation of interaction results, which assumes that the interaction effect cannot be evaluated just by looking at the magnitude or statistical significance of the interaction coefficients alone when the model is nonlinear [40,41]. Thus, we used the lincom command in Stata to obtain combinations of coefficients of the interacted variables in the logistic model and determine the net effects of each variable under both their reference and exposure categories. Evidence of an interaction between locality size and SES on a given risk behavior was based on the variation of the sign, magnitude and statistical significance of the coefficients for the comparison between rural locality (reference category) and the other localities across each SES level.

## Results

**Figure 1:**
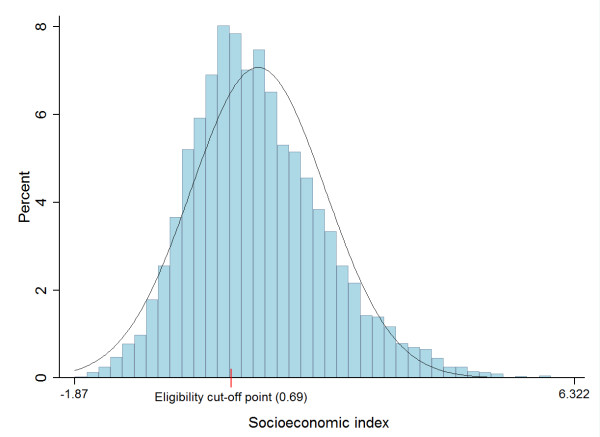
**Socioeconomic index (poverty score) distribution for the entire sample (n = 17,974)**.

The median age for adolescents in this study was 17.5 years. Of the entire sample (n = 17,974), 56% were females, and 42.8% reported an educational level of secondary school. Overall, 20.7% (n = 3,723) resided in 353 rural localities, whereas 34.5% (n = 6,201) were in 221 small semi-urban, 29.4% (n = 5,293) in 90 large semi-urban and 15.3% (n = 2,757) in 40 urban localities. The SES score distribution is shown in Figure 1. In this sample, 62% of adolescents fell under the extreme poverty cut-off point.

Table 1 shows the adolescents' sociodemographic information by gender and locality size. In this sample, approximately 25% of young males and females lived in the poorest households (lowest SES). Only 17% had an educational level of high school, and this same proportion reported being married or in a consensual union. In total, nearly 30% reported that they had smoked, whereas alcohol consumption was reported by 18%. Approximately 8% smoked at the time of the survey. Regarding sexual initiation, 26% had had sexual relations, and among these, less than 30% used a condom at their last sexual encounter. A higher proportion of males than females reported substance consumption and condom use. Further details concerning risk behavior prevalence in this sample can be obtained from other publications [33-38].

**Table 1:** Sociodemographic characteristics of adolescents by locality of residence and gender (n = 17,974)*

		Type of locality according to number of inhabitants							
	**Total**	**Rural**		**Small semi-urban**		**Large semi-urban**		**Urban**	
		**≤ 2,500**		**> 2,500 & ≤ 15,000**		**> 15,000 & ≤ 100,000**		**> 100,000**	
		
		**M**	**F**	**M**	**F**	**M**	**F**	**M**	**F**

Age (Median)	17.5	17.0	17.3	17.4	17.7	17.3	17.7	17.6	17.7
Educational level									
*None*	2.9%	1.2%	2.1%	2.9%	2.4%	3.2%	4.30%	3.0%	3.6%
*Elementary school*	33.5%	32.8%	39.5%	30.6%	33.7%	31.8%	36.4%	32.3%	28.0%
*Secondary school*	42.8%	48.0%	42.0%	42.7%	40.8%	45.3%	39.4%	45.5%	44.2%
*High school*	16.8%	14%	16.2%	19.7%	18.5%	16.5%	15.4%	45.5%	44.2%
*Tecnical or profesional studies/College*	3.7%	1.5%	2.2%	3.7%	4.30%	2.9%	4.3%	5.1%	6.3%
Married/In consensual union	17.2%	7.1%	21.0%	10.4%	23.3%	9.5%	25.6%	10.3%	20.5%
Lowest SES	24.8%	42.2%	43.1%	19.8%	18.1%	24.8%	25.1%	15.3%	12.8%
Ever smoked	27.6%	55.6%	25.8%	39%	15.3%	36.2%	14.8%	35.4%	14.3%
Smokes at present	8.3%	18.4%	2.2%	16.2%	1.9%	14.6%	2.2%	15.8%	2.8%
Drinks alcohol	17.9%	38%	17.6%	25.6%	10.0%	22.3%	9.7%	21.1%	7.7%
Has initiated sexual activity	25.8%	22.6%	27.4%	22.3%	26.9%	20.1%	27.9%	32.2%	29.3%
Condom use on last relation (n = 4,641)	27.7%	52.0%	16.0%	45.3%	11.7%	43.3%	11.2%	60.8%	16.4%
	
	n = 17,974	n = 1,597	n = 2,126	n = 2,678	n = 3,523	n = 2,273	n = 3,020	n = 1,331	n = 1,426

*SES = Socioeconomic status by quartile; M = male; F = female.

The results of multivariate logistic regression analyses considering SES are presented in Table 2. Tobacco and alcohol consumption were significantly associated with locality size, showing a similar trend across all SES levels. Adolescents from larger localities had lower odds of substance consumption compared with adolescents from rural localities. The magnitude of this association was similar for the four SES levels. As shown in Table 2 we did not find a clear and significant association between locality size and being a current smoker, except for the comparison of rural versus urban locality in the 1st quartile and rural versus small semi-urban locality in the 4th quartile.

**Table 2:** Effects of locality size on Mexican adolescents' risk behaviors.

Variable	Ever smoked	Smokes currently	Drinks alcohol	Sexual initiation^c^	Use of condom^d^
Age (continuos variable)	1.15***	1.28***	1.21***	1.65***	0.97
	(1.13-1.17)	(1.24-1.32)	(1.18-1.24)	(1.61-1.69)	(0.92-1.02)
Gender (0 = Female; 1 = Male)					
Single	3.35***	8.04***	2.63***		6.21***
	(3.08-3.65)	(6.69-9.65)	(2.37-2.92)	0.87***	(5.02-7.68)
Married	6.90***	16.36***	8.00***	(0.79-0.95)	2.35***
	(5.69-8.36)	(11.5-23.1)	(6.46-9.90)		(1.84-3.00)
Marital status (0 = Single; 1 = Married)					
Female	0.82***	0.54***	0.51***		0.54***
	(0.71-0.94)	(0.39-0.75)	(0.43-0.60)	N/A	(0.43-0.69)
Male	1.69***	1.11	1.54***		0.20***
	(1.43-2.00)	(0.92-1.33	(1.30-1.83)		(0.16-0.26)
Locality size^b ^according to SES:					
1st Quartile-SES (highest SES)					
Small semi-urban locality	0.41***	0.72*	0.32***	1.07	0.59*
	(0.29-0.58)	(0.51-1.04)	(0.22-0.45)	(0.77-1.49)	(0.34-1.01)
Large semi-urban locality	0.45***	0.77	0.32***	1.42**	0.45***
	(0.31-0.65)	(0.51-1.17)	(0.22-0.46)	(1.01-1.98)	(0.25-0.81)
Urban locality	0.34***	0.58***	0.26***	1.14	0.74
	(0.24-0.49)	(0.39-0.86)	(0.18-0.37)	(0.83-1.57)	(0.44-1.24)
2nd Quartile-SES					
Small semi-urban locality	0.43***	1.05	0.43***	1.48**	0.79
	(0.31-0.58)	(0.74-1.49)	(0.31-0.60)	(1.09-2.01)	(0.44-1.42)
Large semi-urban locality	0.37***	0.98	0.34***	1.40**	0.82
	(0.26-0.53)	(0.67-1.45)	(0.24-0.49)	(1.00-1.94)	(0.44-1.54)
Urban locality	0.33***	0.82	0.34***	1.52**	1.11
	(0.23-0.47)	(0.53-1.26)	(0.24-0.49)	(1.07-2.14)	(0.62-1.98)
3rd Quartile-SES					
Small semi-urban locality	0.38***	0.86	0.37***	0.94	0.72
	(0.28-0.51)	(0.58-1.26)	(0.26-0.54)	(0.70-1.26)	(0.46-1.12)
Large semi-urban locality	0.37***	0.73	0.38***	0.85	0.70
	(0.26-0.53)	(0.47-1.12)	(0.26-0.55)	(0.64-1.13)	(0.45-1.08)
Urban locality	0.33***	0.70	0.25***	1.22	1.00
	(0.22-0.50)	(0.46-1.07)	(0.16-0.39)	(0.92-1.63)	(0.61-1.66)
4th Quartile-SES (low SES)					
Small semi-urban locality	0.41***	0.66**	0.44***	0.82	1.44*
	(0.28-0.59)	(0.45-0.98)	(0.30-0.65)	(0.60-1.12)	(0.99-2.11)
Large semi-urban locality	0.37***	0.71	0.39***	0.69**	1.88**
	(0.25-0.55)	(0.47-1.07)	(0.26-0.58)	(0.51-0.94)	(1.15-3.06)
Urban locality	0.57**	0.78	0.44***	0.95	1.76**
	(0.36-0.90)	(0.54-1.13)	(0.27-0.71)	(0.69-1.33)	(1.06-2.90)
	
	n = 17,974	n = 17,974	n = 17,974	n = 17,974	n = 4,641

Results are odds ratios (IC 95%).

*p ≤ 0.1 **p ≤ 0.05 ***p ≤ 0.01.

a = Analyses carried adjusting by primary sample unit. Multivariate model also includes educational level and survey year (2001 vs 2003). Two interaction terms included: marital status*gender and locality size*socioeconomic status (SES). OR for exposure categories in these variables are calculated with lincom command in Stata.

b = Rural locality of: ≤ 2,500 inhabitants is the reference category comparing to small semi-urban locality: > 2,500 and ≤ 15,000; large semi-urban locality: > 15,000 and ≤ 100,000; and urban locality: > 100,000 inhabitants.

c = Model without marital status*gender interaction term; result is OR for variable gender only.

d = Among those who have initiated sexual activity.

The effect of locality size on sexual behaviors was more complex, with an increased odds ratio (OR) for condom use in larger localities, although only among the poorest group (4th quartile). A different trend was observed for the effect of locality size and sexual initiation across each SES level. However, no significant effects were observed at any SES strata except for the 2nd quartile, in which the odds for sexual initiation were higher among larger localities.

As an alternative form of presenting the results, we have included as supplementary material (Additional File 1) a figure that graphically displays the ORs for the association between locality size and each risk behavior (regardless of significance level) to illustrate the varying magnitude according to SES. In general, the four SES quartiles displayed a similar downward trend for the impact of locality size on substance consumption. However, a moderating effect of SES seemed to take place regarding sexual behavior. In the first two SES quartiles, the odds of sexual initiation were higher in all large settings as compared with rural localities. As poverty increased, the trend for the effects of locality size on sexual initiation adopted a downward trend similar to the one for substance consumption, indicating that, among the poorest youth, living in a semi-urban locality (both small and large) is protective against sexual initiation. However, this protective effect was less evident in large urban localities. Semi-urban localities were associated with lower odds of condom use except for the lowest SES level, indicating that in the poorest stratum, condom use is enhanced by locality size.

Male adolescents exhibited a greater tendency to participate in all of the risk behaviors, and the magnitude of such risk was greater among married adolescents. Being married heightened the risk of having smoked (OR = 1.69; CI 95%: 1.43-2.00) or consumed alcohol (OR = 1.54; CI 95%: 1.30-1.83), and it reduced the possibility of having used a condom at the last sexual encounter (OR = 0.20; CI 95%: 0.16-0.26) as compared with non-married males. For females, being married or in a consensual union reduced the possibility of consuming both tobacco (OR = 0.82; CI 95%: 0.71-0.94) and alcohol (OR = 0.51; CI 95%: 0.43-0.60), being a current smoker (OR = 0.54; CI 95%: 0.39-0.75) and using condoms (OR = 0.54; CI 95%: 0.43-0.69) compared with non-married females (Table 2).

## Discussion

The objective of the present analysis is to identify the role of urbanicity, particularly locality size, as a determinant of risk behaviors among young Mexicans. The results suggest that there is a relationship between adolescents' risk behaviors and the size of the localities in which they live. Furthermore, in the case of condom use, this relationship is moderated by SES. Adolescents' participation in risk behaviors is influenced by the characteristics of their place of residence and context, and particularly for sexual behavior variables, such influence may vary with their household poverty level.

Given the increasing interest in factors related to adolescent and youth wellbeing, these results provide some insights on contextual factors that appear to be related to risk behaviors among Mexican adolescents from low-income areas. Future studies could explore the association between these contextual factors and other aspects of adolescents' wellbeing such as not studying or not working.

These results were obtained from a sample of low-income households in Mexico. Thus, it could be argued that they are valid only for these types of households. However, it is important to highlight that according to official statistics, about half of all Mexicans are living in poverty.

According to the results presented, it seems that the larger the locality size, the less likely adolescents are to consume tobacco or alcohol in comparison with rural settings, and this relation does not seem to be moderated by SES. However, the impact of locality size is less evident regarding sexual behavior. While the data suggest that there is a trend in relation to the effect of locality size on sexual initiation that changes as poverty levels rise, in general no significant effect was observed for the association of locality size and sexual initiation. Residing in a large locality was found to positively influence condom use only among the poorest adolescents, suggesting that in this case, SES does modify the effect of locality size.

The non-significant effect of locality size on sexual behavior can be partially explained by the low prevalence of sexual initiation among adolescents (which translates into a limited number of cases). This has an even greater effect when comparing the different locality sizes and/or SES categories, as stratifying the sample reduces the number of cases further. Nevertheless, our results are in line with prior data showing that residing in an urban locality enhances the possibility of using condoms among Mexican adolescents [31]. However, we found such an association only in one SES strata.

These results appear to be consistent with the proposed hypothesis related to the social and cultural specificities of one's setting. For example, individuals from small rural areas seem to incorporate themselves into adulthood early in life, thus reporting increased participation in non-healthy behaviors. The fact that risk behaviors tend to increase again in larger cities could be related to a higher exposure to incentives to participate in these behaviors, such as higher prevalence in the community and a higher presence of marketing in larger areas [42].

Another possible explanation for the difference in risk behaviors could be that adolescents from rural areas engage more frequently in substance consumption or other risk behaviors as a result of the limited availability of other leisure activities. Also, in rural areas the consumption of alcohol or tobacco may be permissible at social and cultural events, which are often celebrated more frequently because cultural traditions may be stronger in rural areas (compatible with the hypothesis of early adulthood) [43]. What we may be seeing here is a rapid increase in the consumption of substances among rural adolescents that has remained unnoticed, as the majority of studies regarding substance abuse have focused on small samples of urban adolescents.

The results regarding SES seem to indicate that if more resources are available, other aspects such as locality size are less relevant for the sexual behavior variables. That is, resources could reduce the impact of other setting constraints. In our sample, it is among the poorest populations that the size of the locality seems to matter the most.

The present study was conducted using rigorous methodological procedures. However, several shortcomings should be mentioned. First, the information was derived from three different surveys conducted in two separate years, thus involving possible biases associated with tendencies over time and variations in the manner of phrasing the interview questions. To control for these, the analysis was adjusted by measurement year, and we used variables measured in the same way in the three surveys. The questionnaires and application procedures were similar.

The cross-sectional nature of the study poses yet another shortcoming. Given that causality cannot be inferred, the results presented here refer only to associations among variables. The information was self-reported. However, the presence of an adult at the time of the surveys could have affected the self-reporting of risk behaviors. In addition, we did not use any particular statistical test or value to conclude that there was an interaction effect between SES and locality size. Thus, our results should be interpreted cautiously.

The sampling procedures for the surveys may have affected the validity of our results. The sample comprised poor adolescents. Therefore, our findings do not necessarily reflect the behaviors of adolescents in general as they are generalizable only to Mexican adolescents from low-income areas. Furthermore, the samples were selected to represent poor families that could be beneficiaries of the program, which means that this study may not be representative of all adolescents from poor households. For instance, one criterion for eligibility to the program was that the locality had access to health and education. It is possible that adolescents from poor localities were excluded from the sample due to a lack of access to health and education, thus having different risks because they lived in a more isolated context. It is also possible that because of the non-identical sampling procedures there were some differences in criteria used to select households in the analysis for the three surveys. Nevertheless, in the surveys, probabilistic sampling of clusters (localities or blocks) were used to design a sample that would represent areas with a high concentration of poor households in Mexico.

Despite the aforementioned limitations, this study provides valuable and novel information. In the context of limited data on risk behaviors among youths in Mexico, and an increased interest in the topic, this study provides new evidence in this area. The smoking behavior of young people has been a key area of research worldwide and in Mexico in particular [44]. With a trend of increasing prevalence of smoking among adolescents, interventions are being targeted to this group. In addition, because risk behaviors usually reinforce each other, alcohol and drug consumption are also public health concerns regarding adolescents in Mexico, with a vast literature addressing these behaviors and their correlates [42,45-47].

In contrast to previously documented evidence in Mexico [29-31], our findings indicate rural adolescents as a risk population, which is congruent with previous studies in other countries where youth in rural localities exhibit greater rates of consuming tobacco [48], alcohol [49,50] and drugs [20,50] compared with those in urban localities. Although fewer studies have analyzed the relationship between sexual behavior and urbanicity, one study, in line with our results, found no difference between rural and urban adolescents [51].

In Mexico, previous studies have not considered the modulating effect of SES on the association between urbanicity and risk behaviors. Additionally, many studies exploring the influence of urbanicity have established simplistic comparisons of rural versus urban, hindering a proper understanding of their extremely complex relation [49,50] and leading to possibly contradictory results [23,28,51]. According to our results, there is a gradient for the interaction between SES and risk behavior related to locality size, and this gradient does not represent a dichotomy between rural and urban. The risks associated with rural localities in comparison with larger settings exhibit more of a continuum, at least for the categories of localities used in this analysis and the explored variables. These findings provide evidence that may help to explain the variations in risk behavior prevalence across the country and the dynamics of such variations.

The evidence in Mexico has shown that smoking patterns in young men and women are similar [52], whereas in general, women drink less than men [10,53]. As a secondary contribution, this study makes it possible to explore the interaction between marital status, gender and risk behaviors. Married male adolescents are more likely to consume alcohol and tobacco, which has also been demonstrated in other studies in Mexico [54]. In contrast, females who are married or in a consensual union report less substance consumption than their single counterparts. These results are particularly novel in that studies published thus far on substance consumption have scarcely addressed married adolescents. In public health terms, married youths are as important as single youths.

## Conclusions

To our knowledge, this study is the first from Mexico focusing on locality size to explore social determinants of health risk behaviors in youths. According to the present study, adolescents' risk behaviors are related to the size of their localities, and particularly for sexual behaviors, this relation is moderated by SES, indicating that urbanicity has a differential impact related to resources available in the household. Such heterogeneity raises the need for more detailed analyses of both the effects of locality size on health-related behaviors and the responses--also heterogeneous--required to address this situation. More research on this topic is warranted to reinforce youth policy design.

## Competing interests

The authors declare that they have no competing interests.

## Authors' contributions

JPG conceived the idea of the study, designed and supervised the analyses and interpretation of the results, and contributed to the content of the manuscript. EEA was responsible for data management and analysis interpretation and wrote the initial manuscript draft. Both authors revised and approved the final version of the document.

## Pre-publication history

The pre-publication history for this paper can be accessed here:

http://www.biomedcentral.com/1471-2458/11/900/prepub

## Supplementary Material

Additional file 1 **Effects of locality size on adolescents' risk behaviors according to socioeconomic status**. Results are expressed as odds ratios (ORs). Rural locality is the reference category. The model is adjusted by age, gender, marital status, educational level and survey year. Significance levels and ORs for the remaining variables are indicated in Table 2. The 1st quartile corresponds to the highest SES, and the 4th quartile corresponds to the lowest SES (n = 17,974). Click here for file
