# Supplementary material for: Socioeconomic status, urbanicity and risk behaviors in Mexican youth: an analysis of three cross-sectional surveys
Source: BMC Public Health. 2011 Nov 30;11:900. doi: 10.1186/1471-2458-11-900 (PMC3260336; doi:10.1186/1471-2458-11-900)
Supplement: Additional file 1 — Effects of locality size on adolescents' risk behaviors according to socioeconomic status. Results are expressed as odds ratios (ORs). Rural locality is the reference category. The model is adjusted by age, gender, marital status, educational level and survey year. Significance levels and ORs for the remaining variables are indicated in Table 2. The 1st quartile corresponds to the highest SES, and the 4th quartile corresponds to the lowest SES (n = 17,974). [file 1471-2458-11-900-S1.PPT]

## Slide 1
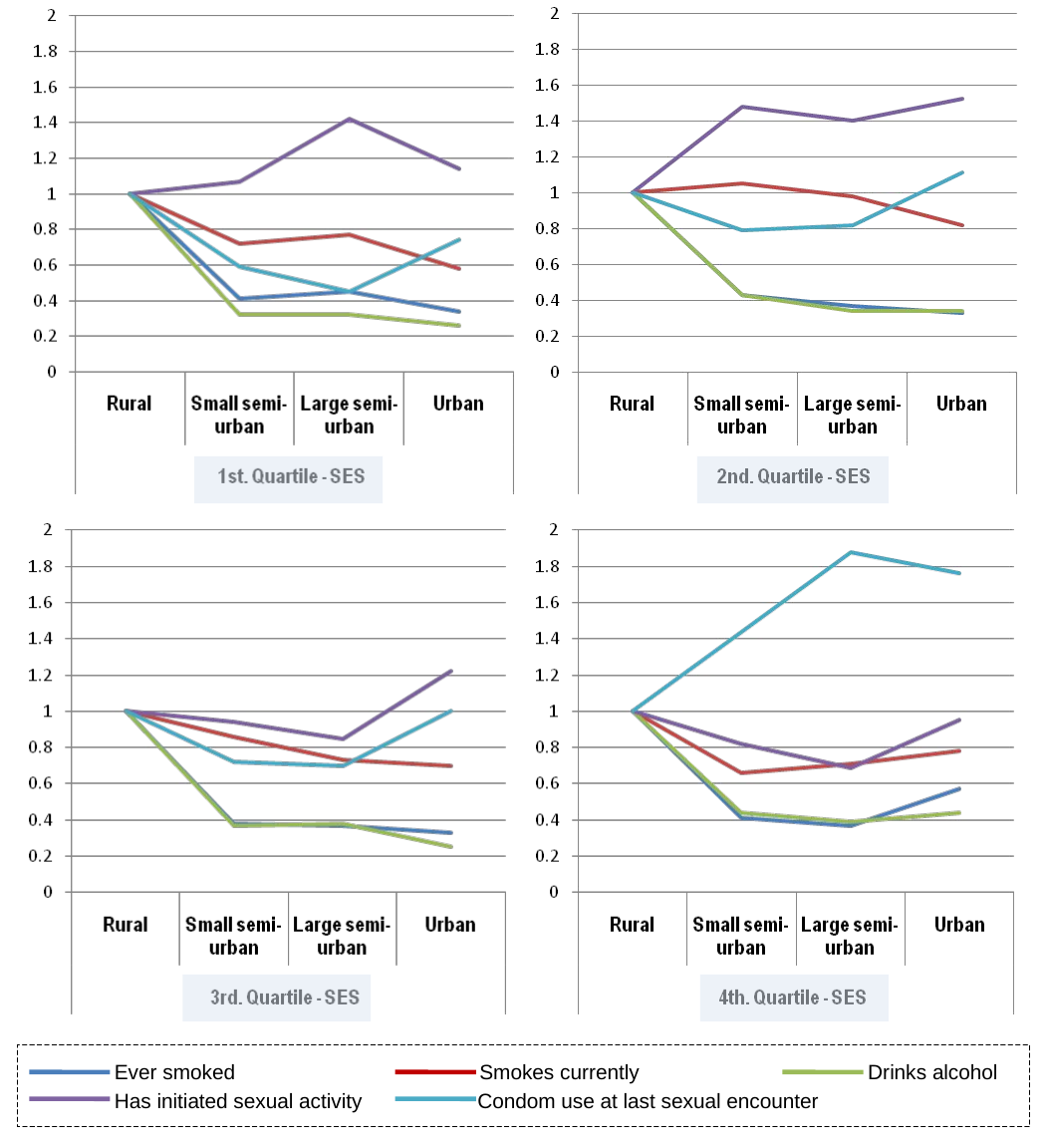

Ever smoked
Smokes currently
Drinks alcohol
Has initiated sexual activity
Condom use at last sexual encounter
